# Inclusion of patient-centered, non-microbiological endpoints and biomarkers in tuberculosis drug trials

**DOI:** 10.3389/frabi.2025.1570989

**Published:** 2025-05-22

**Authors:** Andrew R. DiNardo, Wilbert Sabiiti, Stephen H. Gillespie, Sophia B. Georghiou, Norbert Heinrich, Norbert Hittel, Sami Taghlabi, Danna Carrero Longlax, Mikashmi Kohli, Ursula Panzner, Collins Musia, Christoph Lange, Anca Vasiliu, Rob J. W. Arts, Anna M. Mandalakas, Morten Ruhwald, Lieven J. Stuyver, Reinout van Crevel

**Affiliations:** 1 Department of Internal Medicine and Radboud Center for Infectious Diseases, Radboud University Medical Center, Nijmegen, Netherlands; 2 The Global Tuberculosis Program, Texas Children’s Hospital, Department of Pediatrics, Baylor College of Medicine, Houston, TX, United States; 3 Division of Infectious Disease and Global Health, University of St Andrews, St Andrews, United Kingdom; 4 FIND, Geneva, Switzerland; 5 Division of Infectious Diseases and Tropical Medicine, LMU University Hospital, Munich, Germany; 6 German Center for Infection Research (DZIF), Partner Site Munich, Munich, Germany; 7 Otsuka Novel Products GmbH, Munich, Germany; 8 German Center for Infection Research (DZIF), Partner Site Hamburg-Lübeck-Borstel-Riems, Borstel, Germany; 9 Division of Clinical Infectious Diseases, Research Center Borstel, Borstel, Germany; 10 Respiratory Medicine & International Health, University of Lübeck, Lübeck, Germany; 11 Janssen Global Public Health R&D, Janssen Pharmaceutica NV, Beerse, Belgium; 12 Centre for Tropical Medicine and Global Health, Nuffield Department of Medicine, University of Oxford, Oxford, United Kingdom

**Keywords:** tuberculosis, biomarker, cardiovascular, sequelae, cancer

## Abstract

Tuberculosis drug trials are primarily designed to identify antibiotic regimens with the strongest potency to kill *Mycobacterium tuberculosis*. However, microbiologic cure is not synonymous with improved health and recovery. Beyond antimicrobial efficacy, parameters such as morbidity and mortality related to lung function, cardiovascular health, and cancer should be prioritized. This narrative review emphasizes the critical need to emphasize clinical outcomes as much, if not more, than microbiological endpoints. We examine the underlying pathophysiological mechanisms and determinants of non-microbiological outcomes in tuberculosis, providing a synthesis of current knowledge. While there is growing evidence for some biomarkers to risk stratify TB patients for risk of all-cause mortality, relapse, or lung damage, no evidence was found on TB-associated cancer or cardiovascular disease. In addition to monitoring microbiologic outcomes, clinical trials and treatment cohorts need to capture patient-centered health dimensions more broadly. Finally, we highlight key research gaps and opportunities to evaluate non-microbiological biomarkers, aiming to improve patient monitoring and enable stratified approaches to tuberculosis management.

## Introduction

Tuberculosis (TB) morbidity and mortality arise not only from *Mycobacterium tuberculosis* infection but also from immune dysregulation. Current TB drug development primarily focuses on microbiological endpoints, such as bacillary clearance, to define treatment success (Gillespie et al., 2024). However, these measures fail to capture long-term clinical outcomes, including relapse free cure and post-treatment morbidity and mortality. While antibiotics kill the bacilli, they do not repair immune dysregulation or heal lung dysfunction (Malherbe et al., 2016; Azam et al., 2022; Wallis et al., 2022), which can lead to sterile inflammation, paradoxical reactions, and increased rates of non-communicable diseases and recurrent infections (Critchley et al., 2024; Romanowski et al., 2019; Lee-Rodriguez et al., 2020; Menzies et al., 2021; Vega et al., 2021).

Extrapulmonary TB, such as TB meningitis, TB pericarditis, and spondylodiscitis, highlights the disconnect between microbiological cure and restored health. For instance, TB meningitis is often complicated by severe inflammation, causing stroke, seizures, hydrocephalus, or cranial nerve palsy, with mortality rates of 20–50% during treatment (Soria et al., 2021), and long-term neurological sequelae among survivors (Christensen et al., 2011). However, this review focuses on pulmonary TB, the most prevalent form of the disease with available data.

As part of the UNITE4TB consortium, we recently developed a target product profile (TPP) outlining the ideal characteristics of biomarker tools that would assist with treatment monitoring by quantifying viable *M. tuberculosis* and replacing culture based endpoints (Gillespie et al., 2024). Building on this work, we advocate for the inclusion of non-microbiological endpoints in TB drug trials. This narrative review first outlines the epidemiology of clinical and patient-centered outcomes during and after TB treatment. We then explore the underlying mechanisms of these outcomes, assess putative exploratory biomarkers that reflect these broader health dimensions, and propose a framework for integrating non-microbiological endpoints and biomarkers into TB drug trials to inform comprehensive guideline development.

## Patient-centered outcomes in tuberculosis

The World Health Organization (WHO) estimated that 10.8 million people developed tuberculosis in 2023 (Global Tuberculosis Pragramme W, 2024). The WHO reports an 88% drug-susceptible TB treatment success rate, but this metric is based on completing a full course of antibiotics or achieving bacteriological clearance, defined as smear or culture negativity (Linh et al., 2021). In clinical trials, “cure” is similarly restricted to microbiological endpoints, often assessed at the conclusion of antibiotic therapy and after one or two years, without accounting for patients’ overall health or functional recovery. A more patient-centered approach would prioritize measures of morbidity and mortality outcomes, providing a more holistic appraisal of treatment efficacy.

Microbiological cure does not always equate to restored health. For example, HIV co-infected individuals undergoing anti-retroviral therapy (ART) often experience immune reconstitution inflammatory syndrome (IRIS), a paradoxical reaction driven by sterile inflammation that causes significant morbidity and mortality. However, sterile inflammation and clinical worsening are not exclusive to HIV-associated TB. Estimates suggest that 10–25% of non-HIV-infected patients may also develop paradoxical worsening (**Table 1**), which is usually non-severe, but can be severe including complications, such as hemophagocytic lymphohistocytosis (HLH), a rare but life-threatening inflammatory syndrome (Cheng et al., 2002; Breen et al., 2004; Hawkey et al., 2005; Meintjes et al., 2010; Chahed et al., 2017; Meintjes et al., 2018; Kurver et al., 2024).

**Table 1:** Non-microbiological outcomes during and after completion of TB therapy.

Endpoint	Definition/explanation/ quantification	Frequency during TB	Frequency post TB
All-cause mortality	All-cause, non-traumatic mortality	10–50% of deaths during TB are attributed to non-traumatic, non-TB etiologies (Sterling et al., 2006; Straetemans et al., 2011; Jeong et al., 2023)	SMR of 3.7–7.0 (Fox et al., 2019; Romanowski et al., 2019; Lee-Rodriguez et al., 2020)
IRIS (HIV)	Either unmasking (not previously identified) or paradoxical (previously known) increase in symptoms due to recovery of host immunity after treatment initiation	Occurs in 2–25% of cases depending on definitions used and study settings (Cheng et al., 2002; Breen et al., 2004; Hawkey et al., 2005; Meintjes et al., 2010; Chahed et al., 2017; Meintjes et al., 2018)	Rare; mostly case reports and case series; too few systematic reports; range of 0-2% (Hermans et al., 2024)
Symptomatic paradoxical reactions (non-HIV PR)	Like HIV-associated IRIS, non-HIV PRs are an increase in symptoms due to host immunity in the setting of negative cultures	Occurs in 10–25% of cases depending on definitions applied and study settings (Breen et al., 2004) Occurs less (~2%) in pulmonary TB compared to (25%) extra-pulmonary TB (Hermans et al., 2024)	Range of 0–14%; <1% in pulmonary TB and 3–14% in lymph node TB (Hermans et al., 2024)
Asymptomatic pathologic inflammation	Elevated inflammatory markers after resolution of symptoms	Two to three months during TB treatment, more than half remain hyper-inflammatory (Azam et al., 2022; Wallis et al., 2022)	30–50% TB survivors have elevated inflammation (Ehtesham et al., 2011; Wilson et al., 2018; Azam et al., 2022; Wallis et al., 2022)
Cardio-vascular disease	TB and other infections increase endothelial dysfunction and inflammation, and are associated with increased myocardial infarctions and strokes	Compared to those without TB, people with TB have an IRR of 2.7–3.5 (Critchley et al., 2024)	Survivors of TB have a HR of 2.0 above controls without TB (Salindri et al., 2019b)
Lung dysfunction	Lung destruction and decline in function before, during, and after TB due to bacillary virulence and pathologic inflammation	20% of TB patients with worsening lung dysfunction during successful antibiotic therapy (Plit et al., 1998)	34–74% of TB survivors with abnormal spirometry (Allwood et al., 2021; Menzies et al., 2021) Measured by PET-CT, >80% microbial cured TB have persistent lung inflammation (Malherbe et al., 2016; Odia et al., 2020)
Cancer	Increase risk of cancer during and after tuberculosis	Within first year of TB, SIR 4.7 (95% CI 1.8–12.2) for all cancers and 16.2 (CI 8.6–30.7) for lung cancer (Luczynski et al., 2022)	SIR 1.4 (95%CI 1.3–1.6) years 1–5 after diagnosis for all cancer and 3.0 (95%CI 2.1–4.2) for lung cancer (Luczynski et al., 2022; Moon et al., 2023)
Metabolic dysfunction	Hyperglycemia; pre-diabetes; diabetes; dyslipidemia	~25% TB patients have hyperglycemia, with ~50% resolving with treatment (Menon et al., 2020); >50% of TB patients have increased triglycerides and decreased HDL (Baluku et al., 2024)	AAIR of diabetes 3.8 (95%CI 3.7–4.0) after TB treatment (Salindri et al., 2019b)

SIR, standardized incidence ratio; IRR, incidence rate ratio; AAIR, age adjusted incident rate; HR, hazard ratio; SMR, standardized mortality rate; IRIS, immune reconstitution inflammatory syndrome; PR, paradoxical reaction; TB, tuberculosis; PET-CT, Positron emission tomography- computer tomography; CI, confidence interval.

Persistent inflammation is common despite microbiological cure and studies report elevated systemic and lung-specific inflammation in 50–80% of TB patients following treatment (Malherbe et al., 2016; Odia et al., 2020; Azam et al., 2022). Additionally, significant mortality during TB treatment is often attributed to non-TB-related causes, such as acute myocardial infarctions and strokes, which are markedly increased both during and after therapy (Critchley et al., 2024; Huaman et al., 2015; Salindri et al., 2019b). Non-violent deaths not attributed to tuberculosis account for a substantial proportion of deaths among TB patients in many studies (Sterling et al., 2006; Gillespie et al., 2014; Jeong et al., 2023).

**Table 1** summarizes some key patient-centered, non-microbiological outcomes observed during and after TB therapy. These outcomes emphasize the need for broader endpoints in TB research that reflect not just pathogen clearance but also comprehensive health restoration.

## Post-tuberculosis morbidity and mortality

**Figure 1:**
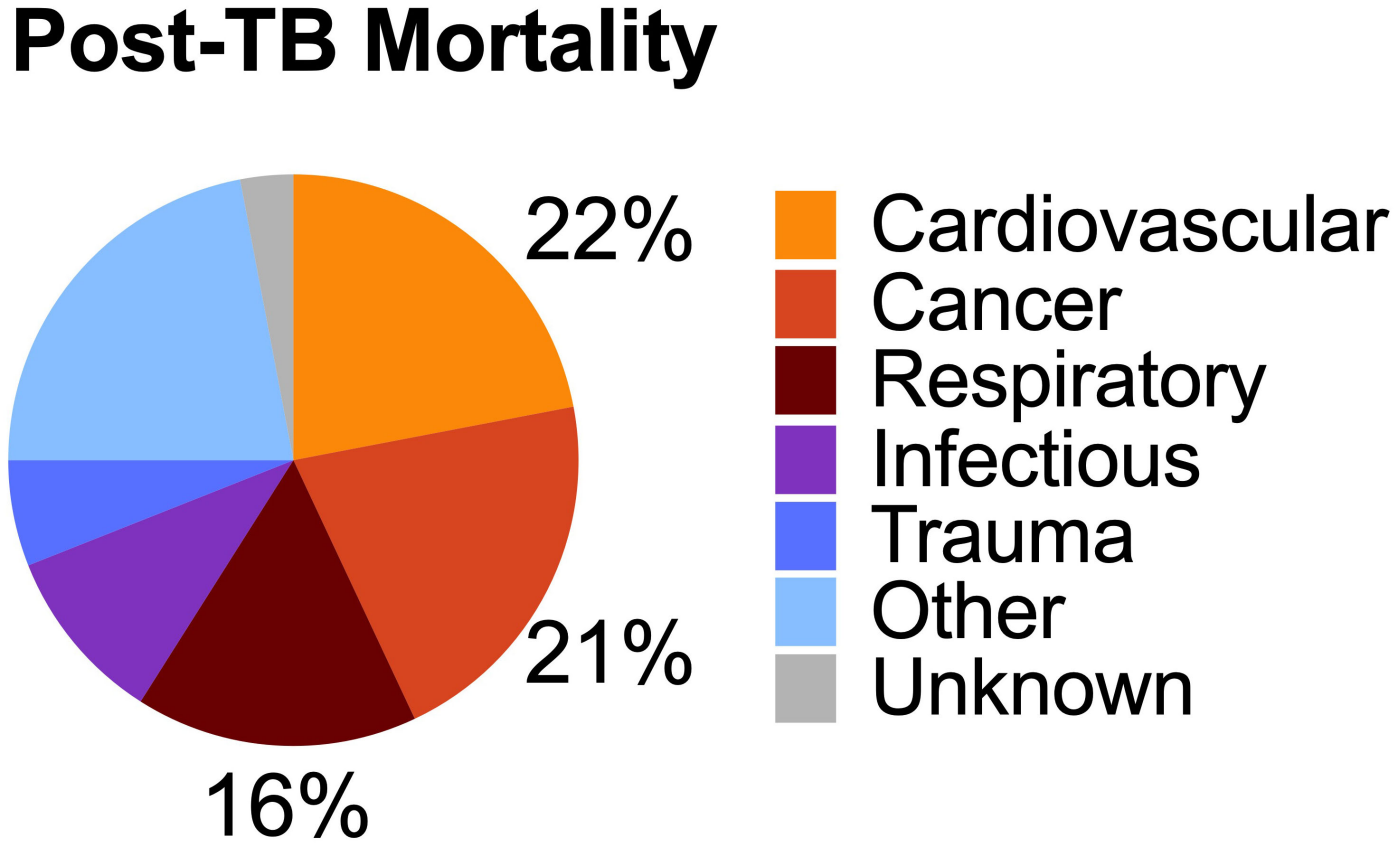
Burden of Post-tuberculosis mortality. Tuberculosis survivors have an increased risk of mortality compared to matched controls. The most common cause of death were cardiovascular, cancer, and respiratory. Source: Based on data from Romanowski et al. (2019).

Despite successful antibiotic therapy, the burden of TB-attributable morbidity extends well beyond treatment completion. Studies estimate that approximately 47% of TB-related disability is experienced in the post-TB period (Menzies et al., 2021). A recent meta-analysis of long-term health describes a 3.7 higher risk of death (95% confidence interval (CI) 3.0–4.6) among TB survivors, compared to matched controls, independent of HIV co-infection (Romanowski et al., 2019). Increased mortality risk is predominantly due to increased cardiovascular, cancer, and respiratory disease among post-TB survivors (**Figure 1**) (Romanowski et al., 2019; Lee-Rodriguez et al., 2020). Similarly, a cohort study in Vietnam reported a standardized mortality ratio (SMR) of 4.0 (95% CI 3.7–4.2) for TB patients compared to the general population (Fox et al., 2019). In the United States, a retrospective cohort study of microbiologically confirmed TB cases revealed an adjusted loss of 7.0 years of life (95% CI 5.5 to 8.4) among TB survivors (Lee-Rodriguez et al., 2020).

Cardiovascular disease is the leading cause of death among TB survivors. A systematic review reported that 20% (95% CI 15–26) of post-TB deaths were due to cardiovascular disease (Romanowski et al., 2019). Additional studies confirm an increased risk of acute coronary syndromes and other cardiovascular events (Chung et al., 2014; Huaman et al., 2015). Malignancy attributable deaths comprise up to 19% of all post-TB deaths (Romanowski et al., 2019), with an incidence risk ratio of 1.9 at 2–4 years after TB treatment compared to controls (Wu et al., 2011). For example, an analysis using the Korean National health and Nutrition Survey found that TB survivors had elevated atherosclerotic cardiovascular disease (ASCVD) risk, one of the most common risk calculators for assessing non-TB cardiovascular disease (Yang et al., 2024).

Despite antimicrobial cure, many TB survivors have persistent respiratory symptoms and decreased quality of life (Mpagama et al., 2021). These respiratory complications are another major source of morbidity among TB survivors. Systematic reviews demonstrate that 10–15% of TB survivors have severe lung impairment, while any form of lung function loss affects 34–74% of TB survivors (Ivanova et al., 2023). Variable presentations include obstructive (18.4–86%), restrictive (16.1–29.7%), or mixed patterns (Ravimohan et al., 2018; Allwood et al., 2020; Khosa et al., 2020; Meghji et al., 2020). Secondary respiratory infections, such as cavitary fungal infections, non-tuberculosis mycobacteria, and recurrent TB are common. In many survivors, chronic inflammation, vascular damage, and lung fibrosis often culminate in post-TB pulmonary hypertension (van Heerden et al., 2024).

The psychological impact of TB persists long after treatment. Stigma, social isolation, income loss, and physical disabilities contribute to a vicious cycle resulting in ongoing mental health challenges. Underlying poverty and catastrophic costs, defined as the loss of at least 20% of annual family income, affect roughly half of TB patients and exacerbate economic and psychological burdens (Bagcchi, 2023). Evidence on post-TB health-related quality of life is mixed, with some studies showing normal scores and others indicating declines in psychological and physical well-being (Muniyandi et al., 2007; Laxmeshwar et al., 2019). The heterogeneity and drivers of normalized or persistent isolation and economic challenges should be evaluated, especially as how this relates to morbidity and mortality.

Modeling studies incorporating post-TB lung disease highlight significant variations in years of life lost, years lived with disability, and disability-adjusted life years (Menzies et al., 2021). Given the complexity and heterogeneity of these various outcomes, a single biomarker seems unlikely to predict the full spectrum of post-TB health challenges.

## Biologic mechanisms

The diverse non-microbiological health outcomes associated with TB are driven by a complex interplay of external factors and biological mechanisms, with immune dysregulation playing a central role. This dysregulation can manifest as anergy (decreased immune responsiveness) and or pathological inflammation. Anergy includes hyporesponsive myeloid cells (immune tolerance) or lymphoid cells (immune exhaustion) (DiNardo et al., 2021). Paradoxically, many TB patients experiencing immune hypo-responsiveness simultaneously with pathologic inflammation (DiNardo et al., 2022). However, the response to TB therapy is highly variable with some TB survivors normalizing inflammatory and immune responsiveness while others experience persistent immune dysregulation.

Although inflammation decreases during TB treatment, a significant proportion of patients exhibit persistent systemic inflammation even after completing successful antibiotic therapy. For instance, studies assessing inflammation via C-reactive protein (CRP) indicate that 30–50% of patients have elevated CRP levels at the end of treatment (Ehtesham et al., 2011; Wilson et al., 2018; Azam et al., 2022; Wallis et al., 2022). Among healthy individuals, elevated CRP and other inflammatory cytokines Interleukin-1 (IL1) and IL6 are associated with increased risks of cardiovascular disease, cancer, and infections (Emerging Risk Factors et al., 2010; Guo et al., 2013; Everett et al., 2020; Khan et al., 2024). Notably, the resolution of hyperinflammation often lags behind the clearance of bacillary load. In one study, only 14% of participants achieved normalized CRP levels at six months of treatment, despite 88% having undetectable bacillary loads (Azam et al., 2022).

In addition to systemic inflammation, TB survivors frequently exhibit persistent lung-specific inflammation. Positron emission tomography-computed tomography (PET-CT) imaging has shown elevated metabolic activity in over 80% of patients following successful TB treatment, highlight the localized inflammatory burden (Malherbe et al., 2016; Odia et al., 2020). Inflammation, a broad and multifaceted phenomenon, is mediated by diverse, redundant, and overlapping molecular pathways. It remains unclear whether different inflammatory drivers necessitate distinct monitoring and management strategies.

**Figure 2:**
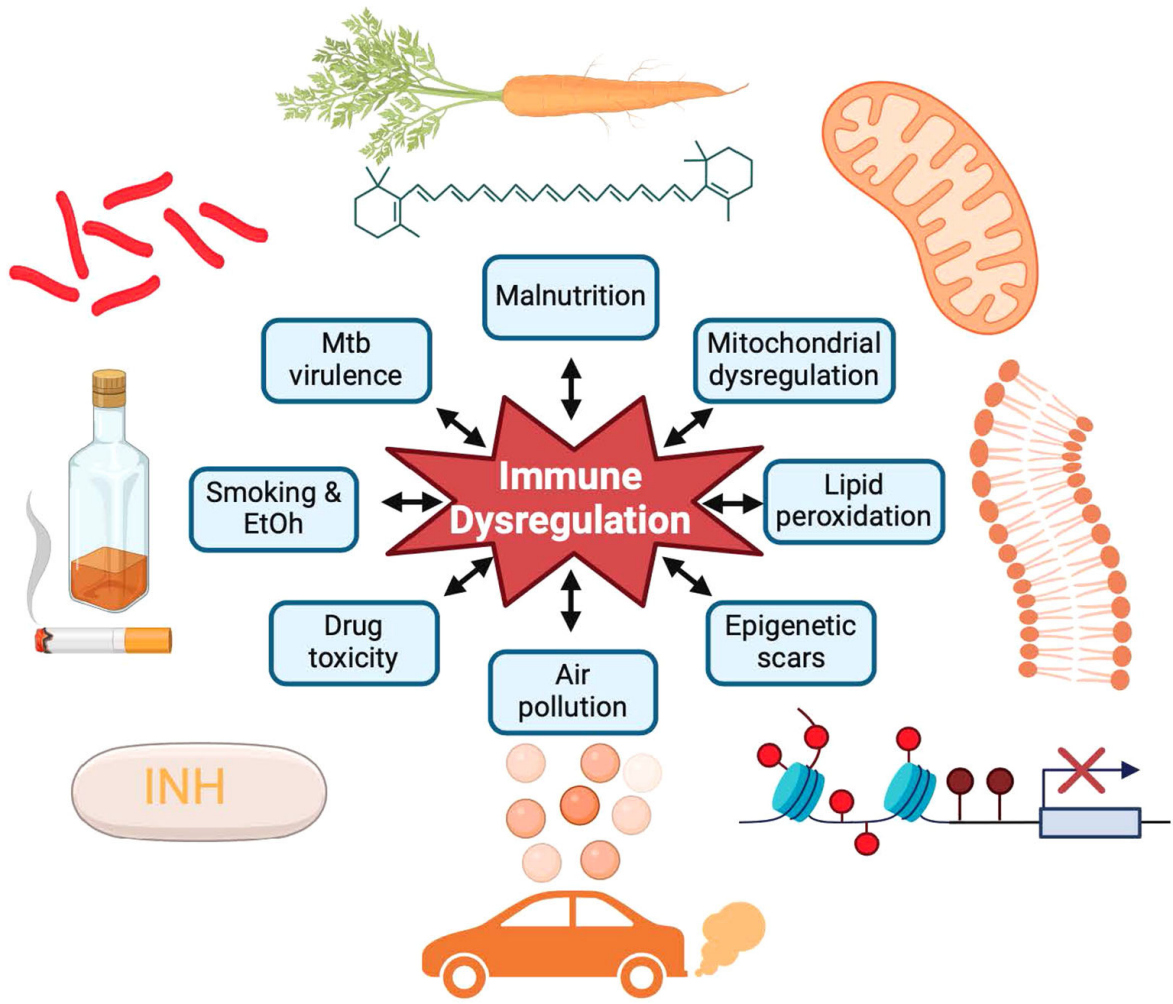
Contributing factors and biologic mechanisms driving tuberculosis morbidity and mortality. Mycobacterial virulence factors, persistent smoking, alcohol (EtOH) use, drug toxicity, malnutrition, and air pollution result in mitochondrial dysfunction, lipid peroxidation, and epigenetic scars resulting in immune dysregulation and inflammation despite successful antibiotic treatment. Source: Created with BioRender.com.

Multiple factors contribute to persistent inflammation and immune dysregulation during and after TB (**Figure 2**). Residual dead bacilli or *M. tuberculosis* antigens that are not fully cleared from tissues can act as chronic immunogens. Persistent inflammation may also arise from malnutrition, micronutrient deficiencies, bronchiectasis with recurrent infections, residual cavities prone to secondary infections, endothelial cell disruption, clonal hematopoiesis, post-TB epigenetic modifications, co-infections, or antibiotic-induced redox imbalances, particularly with isoniazid (Canetti, 1955; Zentner et al., 2020; Allwood et al., 2021; DiNardo et al., 2021; VanValkenburg et al., 2022).

Beyond biological contributors, TB survivors often endure ongoing social and psychological stressors, such as stigma, economic hardships, and social isolation, which can exacerbate systemic inflammation and immune dysregulation. Addressing these multifactorial drivers is critical for improving long-term health outcomes for TB survivors (Green et al., 2023).

Immune dysregulation can also manifest as immune hypo-responsiveness. Depending on the study and definition, approximately 7–35% of successfully treated TB survivors exhibit persistently low immune responsiveness (Delgado et al., 2002; Alexander et al., 2023). This heterogeneity reflects underlying immune dysfunction, with many survivors showing reduced *M. tuberculosis*-specific antigen-induced up-regulation of IL-12, IL-2, IFNγ, diminished antigen-driven proliferative capacity, and heightened expression of immune checkpoint inhibitors and regulatory cytokines such as IL-10 (Boussiotis et al., 2000; Sahiratmadja et al., 2007). Monocytes and myeloid cells also show persistent hypo-responsiveness, with long-term decreased production of IL1β, IL6, and IL12 (Sahiratmadja et al., 2007; DiNardo et al., 2022). Effective host immunity requires a delicate balance that includes generating sufficient pro-inflammatory signals to eradicate the intracellular pathogen without triggering an immune responses that induces collateral tissue damage and increase the risk of cardiovascular disease or lung dysfunction. Some patients suffer from excessive inflammation as well as immune hypo-responsiveness, with TB-associated hemophagocytic lymphohistiocytosis (HLH) as a rare example (Kurver et al., 2024).

Animal and human studies have identified several mechanisms that mitigate immune-induced pathology, including immune checkpoint inhibitors (e.g. PD-1, TIM-3, CTLA-4), inhibitory cytokines (e.g. IL-10, TGF-β), inhibitory metabolites (e.g. itaconate, succinate), and epigenetic immune regulation (Boussiotis et al., 2000; Barber et al., 2011; Nair et al., 2018; DiNardo et al., 2020; Abhimanyu et al., 2024). These pathways likely prevent IRIS and paradoxical reactions during treatment. Future biomarkers must distinguish beneficial immune suppression from detrimental immune inhibition to guide clinical management.

In addition to immune dysregulation, many TB survivors endure tissue destruction that perpetuates inflammation and predisposes them to recurrent infections. Pulmonary cavities often harbor secondary bacterial, fungal and non-tuberculosis mycobacterial infections, compounding inflammation and morbidity (Auld et al., 2024). Residual cicatrization and fibrosis frequently result in restrictive lung impairment, reduced lung volume, and pulmonary hypertension (van Heerden et al., 2024). Other lung complications include tracheobronchial stenosis, bronchiectasis, small airway thickening, parenchymal fibrosis, and pleural thickening. Studies have yet to determine whether the diverse manifestations of lung pathology require a similarly diverse suite of biomarkers for predicting pulmonary outcomes. Current means to monitor TB associated lung damage, not universally available in resource constrained settings, include chest X-rays, spirometry, CT scans, oscillometry, and gas exchange measurements.

Several factors may contribute to immune-dysregulation and tissue pathology in TB. Malnutrition and diabetes increase TB risk and mortality, and are associated with immune dysfunction and tissue pathology. Both human and mycobacterial genetics influence inflammation resolution, and treatment-associated morbidity and mortality. For instance, polymorphisms to human leukocyte antigens or the human matrix mateloproteinase-1 (MMP-1) gene have been associated with increased risk of TB disease risk, development of IRIS, or TB-induced fibrosis (Wang et al., 2010; Tervi et al., 2023; Messah et al., 2024). Similarly, genetic variants regulating inflammasomes correlate with decreased IL-18 and IL-6 levels, and improved forced vital capacity (FVC) (Ravimohan et al., 2020). On the microbial side, evidence from animal models and human studies suggests that *M. tuberculosis* genetics affect disease progression, cavitation, dissemination, and host immune-metabolic responses (Via et al., 2013; Howard et al., 2018). Likely, the interplay between host and mycobacterial genetics shapes inflammation persistence, immune responsiveness, and post-TB sequelae (McHenry et al., 2019).

Unfortunately, tradeoffs exist between achieving microbiologic cure and optimizing overall health. For example, a clinical trial demonstrated that receiving metformin alongside standard antibiotic therapy had slower microbiologic culture conversion rates but showed improved radiographic lung healing (Padmapriydarsini et al., 2021). Moreover certain antibiotics contribute to inflammation. Isoniazid, a cornerstone of TB treatment for over 50 years, produces hydrazine and acetylhydrazine, toxic, reactive metabolites that induces lipid peroxidation and inflammation (Zentner et al., 2020). This mechanism may partly explain the increased cardiovascular risk observed during and after TB.

In summary, persistent immune dysregulation in TB survivors arises from a complex interplay of molecular, social, and anatomic factors, resulting in significant morbidity and mortality (Malefane and Maarman, 2024). Addressing these challenges requires linking these drivers to diverse clinical outcomes, an endeavor that large clinical trial platforms are well-positioned to undertake.

## Putative biomarkers for patient-centered, non-microbiological outcomes

We identified few studies evaluating biomarkers for TB-associated lung damage, all-cause mortality, and relapse. However, no studies specifically addressed biomarkers for TB-associated cardiovascular disease or cancer (**Table 2**).

**Table 2:** Patient-centered, non-microbiological endpoints and putative biomarkers.

Endpoint	Strength of Evidence	Objective Quantification	Putative biomarkers	Unknowns
All-cause mortality	Large studies; few with validation cohorts	3–10 year follow up	MMPs (Walker et al., 2024), albumin (Alvarez-Uria et al., 2013; Okamura et al., 2013; Honjo et al., 2020), Hemoglobin (Isanaka et al., 2012), CRP (Honjo et al., 2020), IL6 (Gupte et al., 2022), TST (Auld et al., 2013; Salindri et al., 2019a), HgA1c (Baker et al., 2011), QFT mitogen (Nguyen et al., 2018; Huang et al., 2019)	Considering the diversity of outcomes, can a few biomarkers risk stratify?
Lung Disease	Moderate; most studies small	Spirometry, 6MWT, SGRQ	IL-18, IL-6 (Ravimohan et al., 2020), TNF, α1-PI, CRP (Kim et al., 2021), CXR (Plit et al., 1998), TGF-β (Christine et al., 2019), IDO (Kim et al., 2021), MMP-1 (Wang et al., 2010; Shanmugasundaram et al., 2022), IL-1β (Wang et al., 2010), FeNO (Maenetje et al., 2024), VEGF (Maenetje et al., 2024), collagen 1a (Baik et al., 2023), transcriptomic signature (Odia et al., 2020), IL-8, cit-H3 (de Melo et al., 2018)	What is the role of exhaled breath condensate? Once identified, how can this be mitigated? Will the same biomarkers identify both obstructive and restrictive lung damage?
Relapse or recurrent secondary infection	Moderate; most small studies; some multi-country studies with validation	3–10 year follow up	IL6 (Gupte et al., 2022), CXCL10 (Mutavhatsindi et al., 2024), HgA1c (Baker et al., 2011), IDO (Kumar et al., 2023), IL22, IL-1β (Mutavhatsindi et al., 2024), LBP (Pillay et al., 2021), MIC (Colangeli et al., 2018), MBLA (Ntinginya et al., 2023)	While microbiology poor predicts relapse, can this be improved with incorporating host immunity? Can existing means to quantify anergy predict recurrence? Can advancing AI radiology help predict secondary mold and NTM infections?
Cardiovascular disease (CVD)	Very poor; no TB-specific CVD biomarker studies identified	3–10 year follow up	No studies have evaluated biomarkers for TB associated CVD	Can existing, non-TB specific, CVD biomarkers or risk calculators accurately predict TB-associated CVD? In non-TB studies, IL-1β, IL6, CRP, GlycA, oxLDL, and HgA1c were associated with CVD risk. Future studies need to evaluate if these are also associated with TB induced CVD risk.
Cancer	Very poor; no TB-specific Cancer biomarker studies identified	3–10 year follow up	No studies have evaluated biomarkers for post-TB cancer risk	Does the biomarker have to be specific to the type of cancer? In non-TB studies, CRP (Guo et al., 2013; Shiels et al., 2015) IL-6 (Tian et al., 2015) were associated with cancer risk. Future studies need to evaluate if these are also associated with TB induced cancer risk.

MMP, matrix metalloproteinases; CRP, C-reactive protein; IL, Interleukin; QFT, QuantiFERON; TST, tuberculin skin test; TNF, tumor necrosis factor; α1-PI, α1 proteinase inhibitor; IDO, indoleamine 2,3-dioxygenase; FeNO, fractional exhaled nitric oxide; VEGF, vascular endothelial growth factor; cit-H3, citrullinated histone H3; CXCL10, C-X-C chemokine ligand 10; MIC, minimal inhibitory concetration; LBP, LPS binding protein; MBLA, molecular bacterial load assay; GlycA, glycoprotein acetyls; oxLDL, oxidized low density lipoproteins.

Most studies on all-cause mortality during TB treatment identified non-specific biomarkers. For example, two large studies, including 466 and 2,854 TB patients, demonstrated that anergy, defined by lower mitogen levels (Huang et al., 2019) or negative QuantiFERON IGRA results (Nguyen et al., 2018), was associated with higher mortality. Similarly, negative TB skin test reactions were linked to increased mortality in two additional large studies (Auld et al., 2013; Salindri et al., 2019a). Other non-specific markers, including anemia, low albumin, lymphopenia, and elevated IL-6 or CRP levels, were also correlated with increased mortality (**Table 2**) (Isanaka et al., 2012; Alvarez-Uria et al., 2013; Okamura et al., 2013; Honjo et al., 2020; Gupte et al., 2022). Interestingly, these studies suggest that both anergy (failure to mount an immune response) and excessive inflammation, increase the risk for death during and after TB.

Research on biomarkers for TB-associated lung disease remains limited, primarily involving smaller studies. For instance, end-of-therapy (EOT) CRP, and α1-protease inhibitor levels were inversely correlated with EOT forced expiratory velocity in the first second (FEV1) (Plit et al., 1998). Elevated TGF levels have been associated with fibrotic lung damage, while proinflammatory cytokines such as IL-6, IL-8, and tumor necrosis factor (TNF) predict necrosis and cavitation (Bekker et al., 1998; Tsao et al., 2000; Lasco et al., 2005; Christine et al., 2019). Persistent neutrophil activation and of inflammasome activation, such as through neutrophil extracellular traps (NETs), have been implicated in lung damage (Ravimohan et al., 2020), highlighting the need for resolving inflammation to facilitate lung healing.

Non-invasive biomarkers for TB-induced lung damage also show promise. For example, measurements of the fraction of exhaled nitric oxide (FeNO) or exhaled breath condensates (e.g. reduced glutathione, lipid peroxidation, and cytokines) identify TB-related lung pathology in smaller preliminary studies (**Table 2**) (Ravimohan et al., 2020; Guzman-Beltran et al., 2021; Mosquera-Restrepo et al., 2022; Maenetje et al., 2024).

TB relapse can be due to recurrence or reinfection. Recurrences, relapses from the same strain, likely results from both residual viable mycobacteria and a deficient immune response. TB reinfection, which occurs at higher risk among individuals with previous TB, likely occurs due to a combination of persistent environmental exposures and persistent immune suppression (Vega et al., 2024). Initial bacillary burden, measured by culture or PCR, is a poor predictor of relapse (Phillips et al., 2016), with a meta-analysis reporting culture having a sensitivity of only 40% for predicting relapse (Horne et al., 2010; DiNardo et al., 2020; Bobak et al., 2022; DiNardo et al., 2022). Quantifying bacterial killing rate may improve relapse prediction (Magombedze et al., 2021), and molecular methods like the Mycobacterial Load Assay (MBLA), which detects viable *M. tuberculosis* RNA, demonstrates initial promise to increase risk of relapse (Ntinginya et al., 2023).

Relapse prediction improves when bacillary burden is combined with additional clinical and immune parameters. For example, a study in South Africa, validated in cohorts in Uganda and Brazil, found that combining culture results, body mass index (BMI), and immune biomarkers (e.g. CXCL10, soluble IL6R, IL12p40, and TNFβ) achieved 75% sensitivity for detecting relapse. Another study involving 130 TB patients from Brazil, Uganda and South Africa reported that combining BMI, culture time to positivity, and biomarkers (e.g. IL1β, CXCL10, IL-22, and complement C3) demonstrated over 80% sensitivity for relapse prediction, a dramatic improvement over culture alone (Mutavhatsindi et al., 2024). Biomarkers of intestinal translocation (e.g. lipopolysaccharide (LPS), LPS-binding protein, soluble CD14) have also been linked to relapse risk (Kumar et al., 2022), with LPS-binding protein confirmed as a strong predictor in a South African cohort (adjusted OR: 8.7, CI 1.2-62.6) (Pillay et al., 2021).

Despite its role as a major cause of TB-associated mortality, no studies were identified evaluating biomarkers for TB-induced cardiovascular disease. Similar to other infections (e.g. HIV, sepsis, pneumonia) (Musher et al., 2019), TB-induced cardiovascular disease likely stems from proinflammatory cytokines (e.g. TNF, IL-6, Il1β) that impair endothelial function, resulting in plaque rupture, and vascular occlusion. Infections also induce dyslipidemia (e.g. elevated low density lipoprotein (LDL), triglycerides, and oxidized LDL) (Baluku et al., 2024), warranting investigation into whether these biomarkers can stratify TB patients at risk for cardiovascular events. Future studies should explore the applicability of traditional cardiovascular risk markers (e.g. hemoglobin A1c, BMI) and identify TB-specific pathophysiological mechanistic drivers of cardiovascular disease.

TB increases the risk of lung cancer (SIR 3.0), esophageal cancer (SIR 2.8), hematologic malignancies (SIR 2.1), and lymphoma (SIR 2.3) (Shiels et al., 2015; Luczynski et al., 2022; Moon et al., 2023). However, we identified no studies evaluating biomarkers for predicting post-TB cancer. Existing cancer biomarkers such as CA19, AFP, or CEA should be explored for TB survivors. Non-TB studies demonstrate that chronic inflammation (elevated IL-6 and CRP) is associated with increased cancer risk (Guo et al., 2013; Shiels et al., 2015; Zhao et al., 2021). Future studies should evaluate the role of IL-6 and CRP in risk stratification for TB associated cancer. TB-related mechanisms, such as epigenetic changes, clonal hematopoiesis, fibrosis, cicatrization, scar formation, immune senescence, and infection induced ROS-mediated gene mutations, likely contribute to cancer development (Hormaechea-Agulla et al., 2021). Identifying these mechanisms could guide biomarker discovery for cancer risk in TB survivors.

Certain non-specific biomarkers could potentially predict multiple poor patient-centered outcomes. For example, pre-treatment IL-6 elevations have been associated with in-treatment mortality, relapse risk, and TB-induced lung damage (Gupte et al., 2022; Khan et al., 2024). Similarly, CRP, a general marker of inflammation, has been linked to cardiovascular disease, cancer risk, lung damage, and recurrent infections in non-TB studies. Low QuantIFERON (QFT) mitogen values, a marker of anergy, correlates with slower culture conversion and higher mortality (Nguyen et al., 2018; Huang et al., 2019; Jacquier et al., 2022). Further research is essential to validate these biomarkers and develop comprehensive strategies for improving patient outcomes beyond microbiological cure.

## Conclusions and recommendations

Despite achieving microbiological cure through successful antibiotic therapy, TB survivors continue to experience significant morbidity and mortality. This includes elevated risks of all-cause mortality, cardiovascular disease, lung dysfunction, cancer, recurrent infections, and diminished quality of life. These outcomes are often overlooked in TB clinical trials due to a historical over-emphasis on microbial endpoints. Unfortunately, there is not yet robust evidence to recommend specific biomarkers. To address these challenges, we propose that clinical trials include host biomarkers in addition to conventional bacterial markers and prioritize patient-centered clinical outcomes. This approach could facilitate the identification of antibiotic regimens with optimal patient-centered outcomes and identify host-directed therapies that effectively eradicate *M. tuberculosis* while minimizing inflammation and reducing long-term morbidity and mortality.

Comprehensive assessment of host and mycobacterial factors, including genetics, nutrition, cytokine levels, epigenetics, and metabolism, alongside extended post-treatment follow-up is critical. However, such efforts are often cost prohibitive for individual clinical trials. Lessons can be drawn from large-scale initiatives such as the UK Biobank and The Cancer Genome Atlas (TCGA), which successfully integrate extensive biomarker data with long-term clinical outcomes. The UK Biobank, supported by a public–private partnership, includes a median of 7 years of follow-up linked to robust epidemiological and genetic data. Similarly, the NIH-funded TCGA has compiled multiomic profiles of more than 40 tumor types from over 30,000 patients. Large clinical trial platforms like UNITE4TB could adopt similar models to establish biobanks addressing multifaceted drivers of TB-associated morbidity and mortality while facilitating access for researchers, via innovative funding mechanisms.

The broad spectrum of TB-related morbidity and mortality underscores the need for more comprehensive research. Current evidence on biomarkers predictive of post-TB outcomes in limited, with few studies examining post-TB lung disease and no studies addressing biomarkers of TB-associated cardiovascular disease or cancer. Given this diversity, a suite of biomarkers is likely necessary to stratify TB survivors based on their risk of adverse outcomes.

Drawing inspiration from the cardiovascular field, research should focus on identifying a core set of clinical and laboratory tests capable of formulating practical risk assessments. Widely used cardiovascular risk calculators such as ASCVD, PREVENT, and the Framingham Risk Score are examples of tools that rely on readily available clinical data. As evidence grows, these scores will need to assess risks specific to country, region, age (pediatric, adult, elderly), HIV, viral load, TB disease location (pulmonary versus extrapulmonary or both) and gender. Similarly, TB research could aim to develop analogous tools to predict and mitigate specific types of TB-associated morbidity and mortality.

In addition to advancing antibiotic regimens, large clinical trial platforms have a unique opportunity to close critical knowledge gaps by identifying biomarkers predictive of the large range of TB-associated morbidity and mortality. By doing so, the field can foster a more comprehensive, patient-centered approach to TB care, ultimately improving long-term outcomes for TB survivors.
