# Correction: Inclusion of patient-centered, non-microbiological endpoints and biomarkers in tuberculosis drug trials

**DOI:** 10.3389/frabi.2025.1748877

**Published:** 2026-03-02

**Authors:** Andrew R. DiNardo, Wilbert Sabiiti, Stephen H. Gillespie, Sophia B. Georghiou, Norbert Heinrich, Norbert Hittel, Sami Taghlabi, Danna Carrero Longlax, Mikashmi Kohli, Ursula Panzner, Collins Musia, Christoph Lange, Anca Vasiliu, Rob J. W. Arts, Anna M. Mandalakas, Morten Ruhwald, Lieven J. Stuyver, Reinout van Crevel

**Affiliations:** 1 Department of Internal Medicine and Radboud Center for Infectious Diseases, Radboud University Medical Center, Nijmegen, Netherlands; 2 The Global Tuberculosis Program, Texas Children’s Hospital, Department of Pediatrics, Baylor College of Medicine, Houston, TX, United States; 3 Division of Infectious Disease and Global Health, University of St Andrews, St Andrews, United Kingdom; 4 FIND, Geneva, Switzerland; 5 Division of Infectious Diseases and Tropical Medicine, LMU University Hospital, Munich, Germany; 6 German Center for Infection Research (DZIF), Partner Site Munich, Munich, Germany; 7 Otsuka Novel Products GmbH, Munich, Germany; 8 German Center for Infection Research (DZIF), Partner Site Hamburg-Lübeck-Borstel-Riems, Borstel, Germany; 9 Division of Clinical Infectious Diseases, Research Center Borstel, Borstel, Germany; 10 Respiratory Medicine & International Health, University of Lübeck, Lübeck, Germany; 11 Janssen Global Public Health R&D, Janssen Pharmaceutica NV, Beerse, Belgium; 12 Centre for Tropical Medicine and Global Health, Nuffield Department of Medicine, University of Oxford, Oxford, United Kingdom

**Keywords:** tuberculosis, biomarker, cardiovascular, sequelae, cancer

An incorrect **Funding** statement was provided. The correct **Funding** statement reads:

“This project has received funding from the Innovative Medicines Initiative 2 Joint Undertaking (JU) under grant agreement No 101007873. The JU receives support from the European Union’s Horizon 2020 research and innovation programme and EFPIA, Deutsches Zentrum für Infektionsforschung e. V. (DZIF), and Ludwig-Maximilians-Universität München (LMU). EFPIA/AP contribute to 50% of funding, whereas the contribution of DZIF and the LMU University Hospital Munich has been granted by the German Federal Ministry of Education and Research”.

The original version of this article has been updated.

