# Supplementary material for: Inclusion of patient-centered, non-microbiological endpoints and biomarkers in tuberculosis drug trials
Source: Front Antibiot. 2025 May 22;4:1570989. doi: 10.3389/frabi.2025.1570989 (PMC12140437; doi:10.3389/frabi.2025.1570989)
Supplement: Supplementary file 1 [file Table1.docx]

**Search Strategy**

To identify putative biomarkers for patient-centered non-microbiologic outcomes, a search was performed using the following databases: PubMed, Embase and SciSpace. Two co-authors screened possible articles. The search spanned from 1990 to 2023, and included terms such as “pulmonary tuberculosis”, “post-tuberculosis lung disease”, “residual lung damage following TB treatment”, “post-tuberculosis cancer biomarkers,” “post-tuberculosis cancer biomarkers”, ”tuberculosis relapse biomarkers”, “inflammatory biomarkers”, “tuberculosis lung function” and “tuberculosis sterile inflammation”. Boolean operators (e.g., “AND”, “OR”) were also used in the search strategy. We included articles with primary data and the most relevant and highest available quality. The search was limited to English language articles. No geographic search criteria were included. When studies provided adequate definitions, good health was defined by being free of cardiovascular disease, cancer, lung damage, and relapse-free longevity.
